# The Impact of Healthcare Professionals’ Characteristics on the Evaluation of Clinical Decision Support Systems: Insights from a Cross-Country Usability and Technology Acceptance Study of the iCARE Tool

**DOI:** 10.1007/s10916-026-02358-5

**Published:** 2026-03-17

**Authors:** Mikko Nuutinen, Anna-Maria Hiltunen, Riikka-Leena Leskelä, Maikki Messo, Anna Salminen, Mari Lahelma, Johanna de Almeida Mello, Anja Declercq, Olena Švihnosová, Kateřina Langmaierová, Daniela Fialová, Federica Mammarella, Rosa Liperoti, Collin Exmann, Hein van Hout, Vanja Pešić, Elizabeth Howard, Agata Stodolska, Katarzyna Szczerbińska, Mor Alon, Ira Haavisto

**Affiliations:** 1 Nordic Healthcare Group Oy, Helsinki, Finland; 2 University of Helsinki, Haartmaninkatu 8, P.O. Box 63, Medicum, Helsinki, 00014 Finland; 3 Department of Industrial Engineering and Management, Aalto University, Helsinki, Finland; 4 Center for Care Research and Consultancy, LUCAS, KU Leuven, Leuven, Flanders, Belgium; 5 Department of Oral Health Sciences, KU Leuven, Leuven, Flanders, Belgium; 6 Department of Social and Clinical Pharmacy, Faculty of Pharmacy, Charles University, Hradec Králové, Czech Republic; 7 Department of Internal Medicine and Geriatrics, 1st Faculty of Medicine, Charles University, Prague, Czech Republic; 8 Fondazione Policlinico Universitario Agostino Gemelli IRCCS, Rome, Italy; 9 Università Cattolica del Sacro Cuore, Milan, Lombardy, Italy; 10 Department of General Practice, Amsterdam UMC, Vrije Universiteit Amsterdam, Amsterdam, the Netherlands; 11 Aging and Later Life Research Program, Amsterdam Public Health Research Institute, Amsterdam, the Netherlands; 12 Boston College School of Social Work, Chestnut Hill, MA USA; 13 The Hinda and Arthur Marcus Institute for Aging Research, Hebrew SeniorLife Institute for Aging Research, Boston, MA USA; 14 Connell School of Nursing, Boston College, Boston, MA USA; 15 Laboratory for Research on Aging Society, The Chair of Epidemiology and Preventive Medicine, Medical Faculty, Jagiellonian University Medical College, Kraków, Poland; 16 Profility Inc, Checker Software Solutions, Haifa, Israel

**Keywords:** Clinical decision support system, Artificial intelligence, Usability, iCARE tool

## Abstract

The development of clinical decision support systems (CDSS) is a complex process requiring early healthcare professional (HCP) involvement. However, engaging HCP is resource-intensive, as reliable assessments often require large participant groups and multiple evaluation rounds. Additional challenge is that HCPs are not a homogenous group, and their characteristics may influence the results that should be considered when designing evaluation studies of CDSS. This study investigated the relationship between HCP characteristics and the ratings of usability and technology acceptance when evaluating CDSS with an AI module. We hypothesize that HCPs who are open to adopting new technology, comfortable with technology, have a higher level of education and are younger will provide more satisfied ratings. The study was conducted with 139 HCPs from seven countries. The evaluated CDSS (iCARE tool) was designed to assist in making treatment decisions for older, multi-morbid patients in home and nursing home settings. HCPs were tasked with deciding whether to continue or discontinue pharmacological and non-pharmacological treatments with the aid of an AI module in the CDSS. Finally, the HCPs completed post-questionnaires evaluating usability and technology acceptance of the CDSS. Data were analyzed using two approaches: statistical modeling (hypothesis-driven) and clustering (data-driven). The statistical models tested the pre-defined hypotheses. Clustering identified participant profiles linking HCP characteristics with the ratings of usability and technology acceptance without any pre-defined hypotheses or assumptions. HCPs who were more open to adopting new technologies or who felt more comfortable with technology evaluated the perceived usefulness of the CDSS higher. Cluster analysis found that prior experience with predictive technologies like the one evaluated in this study was a moderating factor to the satisfaction of the CDSS under evaluation. These findings can inform the design of CDSS evaluation studies, particularly in selecting participant groups to ensure meaningful and representative assessments.

**Supplementary Information:**

The online version contains supplementary material available at 10.1007/s10916-026-02358-5.

## Introduction

Clinical Decision Support Systems (CDSS) are designed to assist healthcare professionals (HCP) in making clinical decisions. Their main purpose is to enhance the quality, efficiency, and safety of patient care by providing recommendations, alerts, and relevant patient data at appropriate points in the clinical workflow [1, 2]. CDSS can support tasks such as diagnosis, treatment planning, medication prescribing, and risk assessment [3, 4, 5, 6, 7]. The implementation of CDSS in a health care system can be a complex and lengthy process including several phases such as design and development, evaluation, integration and maintenance [8]. All phases require or benefit from HCPs involvement. HCPs ensure that the system aligns with clinical workflow and expectations and addresses real-world challenges [9, 10, 11].

This study focuses on the evaluation phase of CDSS, and more precisely on how HCP characteristics influence this phase. The evaluation phase usually encompasses such tasks as system performance or assessing usability, feasibility or technology acceptance [8, 12]. The aim of the evaluation phase is to validate if the CDSS is ready for integration. System performance is typically measured using objective metrics, such as task completion time or the number of correctly identified findings [13, 14, 15, 16]. In contrast, usability, usefulness and technology acceptance are assessed by subjective experiences, commonly measured using validated questionnaire instruments [17, 18, 19].

One challenge to achieve un-biased results in the evaluating phase is that HCPs are not a homogenous group, and their characteristics influence evaluations. Prior studies have indicated that less experienced HCPs tend to benefit more from CDSS support in terms of performance gains, compared to their more experienced counterparts [20, 21, 22]. When usability, usefulness or technology acceptance are measured through subjective rating scales, participants’ characteristics influence how they use question scales. For example, Georgsson et al. [23] measured usability of diabetes health application for 10 patients. They found that younger and more experienced IT/computer users were more satisfied with the application. Raglan et al. [24] found that younger obstetrician/gynecologist were more satisfied information system users than older practitioners. Also, Varonen et al. [25] found that younger physicians had a more enthusiastic attitude toward CDSS than older physicians. Jeddi et al. [26] measured the helpfulness, control and learnability of a health information system with 250 clinicians. They found that experience and age affected helpfulness and learnability negatively and that oppositely the user’s position and education affected helpfulness, usability and learnability positively. Decker et al. [27] and DesRoches et al. [28] found a higher satisfaction with information systems and a higher tendency to use them among young physicians than in their older counterparts. All in all, previous research indicates that younger age, higher education, and experience with technology can influence perceived satisfaction positively when clinical information or decision support systems are evaluated.

The aim of this study was to test prior findings and form new insights on how HCP characteristics influence the use of subjective rating scales in the context of the CDSS evaluation. Our analysis was based on usability and technology acceptance study of an AI-assisted CDSS in a large sample of healthcare professionals (*n* = 139). The contribution of this work is in its practical implications for the design of CDSS evaluation studies, particularly in informing the selection of participant groups to ensure meaningful, representative, and unbiased assessment outcomes.

## Materials and Methods

### iCARE Tool

The iCARE tool is an AI-assisted CDSS developed as part of the EU-Horizon 2020 project “I-CARE4OLD” [29] to support the individualized care of older adults with complex chronic conditions. The core of the iCARE tool is a suite of machine learning models trained and validated on harmonized, multinational datasets derived from the interRAI comprehensive geriatric assessment (CGA) instruments [30] and linked medication and service use data. These models provide real-time predictions of key functional outcomes, including cognitive and functional decline, hospitalization, falls, and expected impact of pharmacological and non-pharmacological interventions. Algorithms behind the models are detailed in [31, 32, 33].

### Participants

The usability and technology acceptance studies were carried out in Belgium, Czechia, Finland, Italy, The Netherlands, Poland, and in the USA. The inclusion criteria for eligibility of HCPs to participate to the study were:

- To be involved in the care process of patients 65 years of age or older with complex chronic conditions in home care or long term care facilities.
- To be qualified health or social care professionals who are in charge of making or proposing therapeutic decisions (e.g., physician, nurse, physiotherapist).
- To have the knowledge and skills needed to be able to interpret the interRAI assessments.
- To have experience with utilizing or to have willingness to utilize InterRAI assessments.

### Study Design

**Fig. 1:**
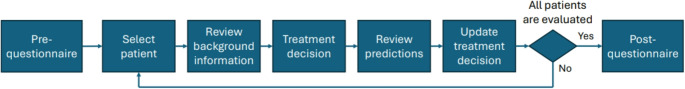
Flowchart of study design. Each participant first filled out a pre-questionnaire, which consisted of background questions and questions related to attitudes towards new technologies. After filling out the pre-questionnaire, participants selected one patient case at time, reviewed summary information and interRAI assessments of that patient and made treatment selection. After that, the risk predictions and treatment effect estimations for the patient calculated by the iCARE tool were presented and participants could update their treatment selections. Finally, when the participants had reviewed all patient cases, they answered post-questionnaires: Technology Acceptance Model (TAM) [18] and Post-study System Usability Questionnaire (PSSUQ) [35]. Participants also answered additional questions about how they perceived the potential value of the tool and if they felt that the tool raised legal or ethical issues

Figure 1 illustrates the flowchart of the study design. The study protocol is detailed in [34]. Each participant first completed a pre-questionnaire (Table S1, Figure S1), which included socio-demographic background information as well as questions regarding education, profession, current position, and professional experience. In addition, the pre-questionnaire contained five items assessing participants’ experiences with and attitudes towards new technologies (Table S2).

After completing the pre-questionnaire, participants reviewed the background information for the selected patient cases (Figure S2). This information comprised both summary data (Figure S3) and the patients’ interRAI assessments. Participants were then asked to make an initial treatment decision (Figure S5a), followed by a revised decision (Figure S5b) after being presented with the risk predictions and treatment effect estimations generated by the iCARE tool (Figure S4). Finally, after reviewing all patient cases, participants completed the post-questionnaires.

### Post-Questionnaires

The post-questionnaires consisted of the Technology Acceptance Model (TAM) [18], the Post-Study System Usability Questionnaire (PSSUQ) [35], and four additional questions (Q1–Q4). The additional questions focused on the potential value of the tool and whether it raised any legal or ethical concerns (Table S5).

The TAM evaluates an individual’s intention to adopt a new technology, based on two key constructs: perceived usefulness (PU) and perceived ease of use (PEU). Both PU and PEU include six items (Table S3), with responses rated on a scale ranging from “Extremely likely” to “Extremely unlikely.” TAM is widely regarded as a general framework for assessing technology acceptance and has been extensively applied in studies of healthcare professionals’ willingness to adopt new tools. [36, 37, 38]

The PSSUQ provides a standardized measure of software usability and was originally validated to differentiate between difficult and easy-to-use systems. It employs a 7-point Likert scale, where lower scores indicate higher perceived usability. In this study, 16 PSSUQ items were applied (Table S4), yielding four component scores: overall usability (all items), system quality (items 1–6), information quality (items 7–12), and interface quality (items 13–15). PSSUQ has been widely used in usability evaluations across diverse contexts. [39, 40, 41]

All patient cases, the questionnaires, and the user interface of the iCARE tool were originally in English but were translated into the native language of each participating country by two independent interpreters, and then translation differences were solved by a clinician acknowledged with interRAI tools and medical terms used specifically in geriatrics and gerontology.

### Ethical Considerations

Ethical approvals of Ethics Committees were granted in each country (except Finland) before the pilot experiment [34]. In Finland, the organizations’ research permits were sufficient to conduct the pilot. In addition, all participants gave their informed consent to participate in the study.

### Statistical Methods

The findings were based on two analysis types. In the first analysis, we tested four hypotheses. The hypotheses were derived from prior research. The hypotheses were:

- H1: HCPs who are more open to adopting new technologies are more satisfied with the tool.
- H2: HCPs who are more comfortable with technology are more satisfied with the tool.
- H3: Younger HCPs are more satisfied with the tool.
- H4: HCPs with higher education are more satisfied with the tool.

The hypotheses were tested using regression models, adjusted for age, gender, education and years with use of RAI tools for H1 and H2, gender, education and years with use of RAI tools for H3 and age, gender and years with use of RAI tools for H4. The outcomes of the regression models were the responses of the TAM PU and PEU and PSSUQ composite scores. Statistical significances were determined using Holm-Bonferroni-adjusted p-values to control for multiple comparisons and reduce the risk of Type I errors [42]. The internal consistency of the question scales was assessed using Cronbach’s Alpha [43, 44].

In the second analysis, we conducted a cluster analysis. Clustering was used as a data-driven technique to seek insights from the data without predefined assumptions. First, latent dimensions for TAM and PSSUQ questionnaires were determined by principal component analysis (PCA) [45]. Then, we calculated the clusters from datapoints expressed by the latent dimensions using the K-means algorithm [46, 47]. Sampling adequacy of the variable set and each individual variable for the PCA analysis was measured by the Kaiser-Meyer-Olkin (KMO) measure [48]. The suitability of the data for PCA was tested with the Bartlett’s test of sphericity measuring adequate correlations between variables [49]. One challenge in the PCA and the cluster analyses is to choose the number of principal components and clusters for the final solution. In this study we used the measures of eigenvalues in the scree plot to search for the optimal number of principal components. Average silhouette width [50] was used to search for an optimal number of clusters. Eigenvalues correspond to the amount of variation explained by each component, that is, how much information each component contains [51, 52]. The average silhouette width measures compactness and separation of the clusters. Differences between the members of the identified clusters were analysed using the mean scores of the questionnaires. A difference was deemed statistically significant when the Holm–Bonferroni–adjusted p-value was < 0.05. After cluster analysis, post-hoc analyses were conducted to test the significancy of the found moderator variables and examining the effect of clinical experience on the clinical value of the tool.

Data analyses were conducted using Python (version 3.13.0). Models for regression analyses (hypothesis testing and post-hoc analyses) were calculated using statsmodels package (version 0.9.0). PCA analysis was calculated using sklearn package (version 1.6.0).

## Results

### Pre-Questionnaires: Participants

A total of 167 clinicians participated in the experiment. Of these, 28 were excluded due to missing identification information or protocol violations during the experiment. Consequently, the final analysis included data from 139 clinicians (Table 1). The mean age was 46.3 years (SD = 11.1). Women comprised 72.7% of the participants. Among the participants, 54.7% were physicians, and 20.9% held a doctoral degree. A total of 51.0% had prior experience using a decision support system (DSS), while 23.0% had previously used a DSS with a predictive component.

### Post-Questionnaires: Usability and Technology Acceptance

The boxplots (median scores, quartiles and complete ranges) of the TAM and PSSUQ questions (Table S3 and S4) for the iCARE tool are provided in Figures S6-S7. The Cronbach’s alpha values of the subscales were between 0.823 and 0.949, indicating good to excellent reliability. The average scores of TAM (technology acceptance) Perceived Usefulness subscale was 3.38 (SD 1.63) and Perceived Ease of Use 2.62 (SD 1.66). A value of 2 means that the statement is “Quite likely” and value of 3 “Slightly likely”.

The average score of PSSUQ (usability) system quality subscale was 2.56 (SD 1.51), information quality 3.50 (SD 1.74) and interface quality 3.07 (SD 1.67) (Figure S8). The scale is from 1 (strongly agree) to 7 (strongly disagree). The benchmarking values of the PSSUQ subscales according to the research of [53] are 2.80 (System quality), 3.02 (Information quality) and 2.49 (Interface quality). This means that the iCARE tool was evaluated better than the benchmark for system quality and worse for information and interface quality.

**Table 1:** Participants of the study

Item	All	US	FI	NL	IT	BE	CZ	PL
Clients, n	139	20	20	21	20	19	19	20
Age, mean (std)	46.33 (11.14)	51.55 (10.28)	45.0 (9.03)	38.81 (9.24)	48.45 (6.91)	47.84 (10.4)	46.68 (15.84)	46.45 (11.66)
Gender female, n (%)	101 (72.66%)	19 (95.0%)	19 (95.0%)	14 (66.67%)	9 (45.0%)	15 (78.95%)	8 (42.11%)	17 (85.0%)
Home care, n (%)	63 (45.32%)	10 (50.0%)	11 (55.0%)	11 (52.38%)	10 (50.0%)	10 (52.63%)	1 (5.26%)	10 (50.0%)
Long term care, n (%)	76 (54.68%)	10 (50.0%)	9 (45.0%)	10 (47.62%)	10 (50.0%)	9 (47.37%)	18 (94.74%)	10 (50.0%)
Position - nurse, n (%)	52 (37.41%)	16 (80.0%)	15 (75.0%)	4 (19.05%)	0 (0.0%)	10 (52.63%)	0 (0.0%)	7 (35.0%)
Position - physician, n (%)	76 (54.68%)	2 (10.0%)	2 (10.0%)	17 (80.95%)	20 (100.0%)	5 (26.32%)	18 (94.74%)	12 (60.0%)
Position - other, n (%)	11 (7.91%)	2 (10.0%)	3 (15.0%)	0 (0.0%)	0 (0.0%)	4 (21.05%)	1 (5.26%)	1 (5.0%)
Degree - basic, n (%)	8 (5.76%)	4 (20.0%)	4 (20.0%)	0 (0.0%)	0 (0.0%)	0 (0.0%)	0 (0.0%)	0 (0.0%)
Degree - bachelor, n (%)	21 (15.11%)	5 (25.0%)	13 (65.0%)	1 (4.76%)	0 (0.0%)	2 (10.53%)	0 (0.0%)	0 (0.0%)
Degree - master, n (%)	81 (58.27%)	8 (40.0%)	3 (15.0%)	17 (80.95%)	9 (45.0%)	12 (63.16%)	19 (100.0%)	13 (65.0%)
Degree - doctor, n (%)	29 (20.86%)	3 (15.0%)	0 (0.0%)	3 (14.29%)	11 (55.0%)	5 (26.32%)	0 (0.0%)	7 (35.0%)
Degree - high*, n (%)	110 (79.14%)	11 (55.0%)	3 (15.0%)	20 (95.24%)	20 (100.0%)	17 (89.47%)	19 (100.0%)	20 (100.0%)
Years in clinical practice, mean (std)	18.78 (11.92)	24.65 (13.0)	15.7 (8.35)	12.29 (9.07)	18.2 (6.33)	20.74 (11.71)	23.21 (17.44)	17.3 (11.35)
Working experience with interRAI, n (%)	68 (48.92%)	9 (45.0%)	20 (100.0%)	3 (14.29%)	20 (100.0%)	13 (68.42%)	1 (5.26%)	2 (10.0%)
Years with use of RAI tools, mean (std)	5.0 (7.19)	3.5 (6.19)	7.95 (6.24)	1.0 (3.07)	15.25 (7.84)	4.05 (5.71)	0.68 (1.0)	2.5 (5.38)
Comfortable with using technology^1^, mean (std)	3.89 (1.14)	3.75 (1.52)	3.9 (1.33)	3.24 (1.0)	4.25 (0.85)	4.05 (0.85)	4.21 (1.27)	3.9 (0.79)
Open adopting new technologies^2^, mean (std)	4.2 (0.96)	4.65 (0.49)	4.0 (1.21)	3.62 (0.92)	4.6 (0.6)	4.05 (0.71)	4.0 (1.41)	4.5 (0.69)
Have used DSS in work^3^, n (%)	71 (51.08%)	9 (45.0%)	18 (90.0%)	12 (57.14%)	3 (15.0%)	6 (31.58%)	14 (73.68%)	9 (45.0%)
Have used predictions^4^, n (%)	32 (23.02%)	6 (30.0%)	3 (15.0%)	10 (47.62%)	4 (20.0%)	2 (10.53%)	6 (31.58%)	1 (5.0%)

*US* USA, *FI* Finland, *NL* Netherlands, *IT* Italy, *BE* Belgium, *CZ* Czechia, *PL* Poland, *DSS* decision support system, *std* standard deviation

*: master or doctor degree

1: How comfortable are you with using technology in general in your daily professional activities? 1 (Not comfortable at all) 2 3 4 5 (Very comfortable)

2: How open are you to adopting new technologies in general in your healthcare practice? 1 (Not open at all) 2 3 4 5 (Very open)

3: Have you used any decision support systems in your work? No/Yes

4: Have you used any decision support systems with predictions for patient trajectories? No/Yes

### Hypothesis Tests

Table 2 presents parameters (95% CI) of the regression models for the exposure variables “Openness to adopting new technology” (H1), “Comfortable with technology” (H2), “Age” (H3) and “Education” (H4) for the PU and PEU outcomes of TAM and system quality, information quality, interface quality and overall quality components of PSSUQ. Tables S6 and S7 presents parameters for all individual questions of the TAM and PSSUQ questionnaires. Based on the results, we can accept hypotheses H1 and H2. If participants perceived themselves as open to new technology or were more comfortable with technology, they were more satisfied with the usability of the iCARE tool (measured by PSSUQ instrument) and reported higher levels of technology acceptance (measured by TAM instrument). The results do not fully confirm the hypotheses H3 or H4. The directions of the signs of the parameters for younger age or a higher education were mostly consistent with the hypotheses, but the p-values were not significant.

**Table 2:** Hypothesis tests. Model H1: Regression models of openness to adopting new technology” **. Model H2: Regression models of comfortable with technology***. Model H3: Regression models of younger age. Model H4: Regression models of higher education. The models H1 and H2 were adjusted by age, gender, education and years with use of RAI tools. The model H3 was adjusted by gender, education and years with use of RAI tools and H4 age, gender and years with use of RAI tools

Question	Model H1 parameter	95% CI	*p*-values	Model H2 Parameter	95% CI	*p*-values
TAM PU subscale	-0.614	[-0.85, -0.377]	< 0.001*	-0.379	[-0.602, -0.156]	0.001*
TAM PEU subscale	-0.584	[-0.79, -0.378]	< 0.001*	-0.414	[-0.609, -0.219]	< 0.001*
PSSUQ system quality	-0.384	[-0.598, -0.169]	< 0.001*	-0.286	[-0.485, -0.086]	0.005*
PSSUQ information quality	-0.31	[-0.542, -0.078]	< 0.009*	0.004	[-0.21, 0.219]	0.967
PSSUQ interface quality	-0.484	[-0.686, -0.282]	< 0.001*	-0.218	[-0.412, -0.025]	0.027
PSSUQ Overall	-0.381	[-0.564, -0.198]	< 0.001*	-0.16	[-0.334, 0.014]	0.072
	Model H3 Parameter	95% CI	*p*-values	Model H4 Parameter	95% CI	p-values
TAM PU subscale	0.007	[-0.014, 0.029]	0.512	0.033	[-0.613, 0.679]	0.920
TAM PEU subscale	0.012	[-0.007, 0.031]	0.210	-0.318	[-0.889, 0.254]	0.273
PSSUQ system quality	0.011	[-0.008, 0.03]	0.251	-0.588	[-1.148, -0.027]	0.040
PSSUQ information quality	0.006	[-0.014, 0.026]	0.555	-0.119	[-0.714, 0.475]	0.692
PSSUQ interface quality	0.011	[-0.007, 0.029]	0.243	-0.272	[-0.817, 0.273]	0.326
PSSUQ Overall	0.009	[-0.007, 0.025]	0.273	-0.333	[-0.819, 0.152]	0.177

*Significance level of Holm-Bonferroni-adjusted p-value < 0.05

** How open are you to adopting new technologies in general in your healthcare practice? 1 (Not open at all), 2, 3, 4, 5 (Very open)

*** How comfortable are you with using technology in general in your daily professional activities? 1 (Not comfortable at all), 2, 3, 4, 5 (Very comfortable)

*TAM* technology acceptance model

*PU* perceived usefulness

*PEU* perceived ease of use

*PSSUQ* post-study system usability questionnaire

*CI* confidence intervals

### Clustering

All TAM and PSSUQ questions were used as variables for the PCA (Table S8). The KMO coefficient for the variable set was 0.89, and for each individual variable 0.77–0.95, indicating good sampling adequacy [54]. Bartlett’s test of sphericity was significant *p* < 0.001, indicating that the correlation matrix for the variable set was suitable for the PCA. Figure S9 shows the explained variance for the 10 first principal components (scree plot). The ‘knee’ in the scree plot can be noticed after three or four components. We selected four components for the further analysis. These four principal components explained 44.2%, 12.7%, 9.1% and 4.4% of the variation in the data set. The data points presented in the space of the four principal components were clustered by the K-means algorithm. The number of clusters was selected based on the Silhouette score (Figures S10). The highest value is for the clustering solution of three clusters. The center values of the principal component for the clusters are presented in Figure S11.

The numbers of participants in clusters one (C1), two (C2) and three (C3) were 84, 34 and 21, respectively (Table 3). The primary differences were in the characteristics of “Comfortable with using technology”, “Open to adopting new technologies”, “Have used DSS or predictions in work”, education, and position. The characteristics of the clusters were determined by comparing the distributions and the means of the variables across the clusters (Table 3). The clusters were profiled as:

- C1: Have less prior experience with DSS or prediction tools in clinical work, and more often have working experience with interRAI.
- C2: Comfortable with using technology and open to adopting new technologies. More often male than in other clusters. More often physician than in other clusters. Have used DSS and prediction tools in clinical work.
- C3: Less comfortable with using technology or open to adopting new technologies. Longer experience with older adults.

**Table 3:** Characteristics of participants in different clusters

Item	All	C1	C2	C3	*P*-value (C1 vrs. rest)	*P*-value (C2 vrs. rest)	*P*-value (C3 vrs. rest)
Clients, n	139	84	34	21			
Age, mean (std)	46.33 (11.14)	46.11 (10.24)	45.35 (12.19)	48.81 (12.94)	0.985	0.434	0.363
Gender female, n (%)	101 (72.66%)	65 (77.38%)	19 (55.88%)	17 (80.95%)	0.173	0.015	0.435
Long term care, n (%)	76 (54.68%)	39 (46.43%)	25 (73.53%)	12 (57.14%)	0.023	0.017	1.000
Position - physician, n (%)	76 (54.68%)	41 (48.81%)	25 (73.53%)	10 (47.62%)	0.117	0.017	0.488
Degree - high, n (%)	110 (79.14%)	60 (71.43%)	30 (88.24%)	20 (95.24%)	0.006	0.153	0.076
Years in clinical practice, mean (std)	18.78 (11.92)	18.23 (11.1)	18.0 (12.32)	22.24 (14.25)	0.755	0.486	0.206
Years of experience with older adults, mean (std)	16.49 (10.69)	16.75 (10.65)	14.12 (10.46)	19.29 (10.91)	0.674	0.104	0.169
Working experience with interRAI, n (%)	68 (48.92%)	48 (57.14%)	10 (29.41%)	10 (47.62%)	0.024	0.010	1.000
Years with use of RAI tools, mean (std)	5.0 (7.19)	5.46 (7.07)	3.97 (7.43)	4.81 (7.43)	0.135	0.075	0.926
Comfortable with using technology, mean (std)	3.89 (1.14)	3.79 (1.11)	4.41 (0.89)	3.48 (1.36)	0.077	0.001	0.107
Open adopting new technologies, mean (std)	4.2 (0.96)	4.19 (0.92)	4.44 (0.86)	3.86 (1.2)	0.578	0.049	0.108
Have used DSS in work, n (%)	71 (51.08%)	37 (44.05%)	24 (70.59%)	10 (47.62%)	0.056	0.010	0.815
Have used predictions, n (%)	32 (23.02%)	13 (15.48%)	14 (41.18%)	5 (23.81%)	0.013	0.009	1.000

Abbreviations: *RAI* resident assessment instrument, *DSS* decision support system, *std* standard deviation

## Discussion

This study investigated how healthcare professional characteristics influence the responses in subjective evaluation instruments used for the usability and technology acceptance assessment of a CDSS with an AI module. Firstly, the hypothesis-based analysis indicated that HCPs who are more open to adopting new technologies or who feel more comfortable with technology tend to evaluate the usefulness of the CDSS higher. This is in line with prior literature indicating that positive attitudes toward technology are associated with higher satisfaction of clinical information systems [23]. Hypotheses related to demographic factors of age or education were not supported in the statistical analysis, despite the fact that prior research has found that younger and more educated clinicians are more satisfied with clinical tools [24, 25, 27, 28]. However, the results of this study did not contradict these hypotheses either; the directions of the coefficients of the models were in line with prior research, but without statistical significance.

The cluster analysis based on the post-questionnaires (TAM and PSSUQ) resulted in three distinct clusters, and provided additional insights from the data. HCPs in cluster C1, who had limited experience from using CDSSs were the most satisfied with the iCARE tool and would recommend it to colleagues. However, our results indicate that HCPs’ prior experience with similar predictive technologies significantly influenced their satisfaction of the iCARE tool. This is apparent in cluster C2, which included HCPs comfortable with technology and with earlier experience with predictive tools. Members of C2 evaluated the information quality of the iCARE tool more critically. It seems that previous experience may form reference levels against which new systems are then compared.

In the post-hoc analysis, we further examined the effect of clinical experience on the perceived clinical value of the iCARE tool. The analysis indicated that HCPs with more years of clinical experience rated the iCARE tool lower in terms of whether it could provide valuable information to support the care path. However, the absence of differences in perceived support for work, ethical or legal concerns, and willingness to recommend the tool showed that more experienced HCPs did not consider the iCARE tool overall to be more problematic or less useful, apart from its perceived support for the care path. This may be related to the fact that experienced HCPs already have substantial clinical knowledge and intuition, and therefore perceived the tool as offering less novel information, particularly for care path planning, compared to less experienced HCPs.

### Practical Implications

Information about how HCP characteristics may influence satisfaction with CDSS can be utilized (1) during the development phase of CDSS and (2) during the training phase of CDSS implementation. In the development phase, this information could be used for selecting participants to test and give feedback on the system. If the profile of the system’s end user is known, the test participants should match this profile, or the results should be weighted accordingly to minimize bias in the findings. A clear result was that the openness to adopting new technology affected how the system was perceived and rated. Therefore, in selecting participants, individuals should be chosen to represent a balanced range across the openness to adopting scale. This factor is further influenced by what is known as participation or non-response bias [55], meaning that clinicians with a more positive attitude towards technology or digital applications are more likely to volunteer for such studies.

Another implication of our results is that one should include healthcare professionals with prior experience of similar tools into the testing group. This is because they seem to be more critical of its usefulness, even if they are open to adopting new technology. This counterbalances the “hype” affecting the ratings given by HCPs who are excited about new technologies, but without prior practical experience cannot put the CDSS being evaluated into context. In addition to previous use, it might be worth asking about satisfaction with previous use. Another bias may be due to previous bad experiences, which makes users reluctant to use the CDSS.

Although the results of this study did not fully support previous findings that education and age influence perceived satisfaction, taking these factors into account in the study design may still be important. Age and education may be related to how well a user is able to use or adopt information systems. More highly educated and younger individuals are possibly more experienced with computers and information systems and, as a result, may find new tools more approachable [56]. Based on this, participants with lower educational backgrounds and older individuals may require more training and support during testing and implementation of the system.

### Strength and Limitations

One strength of the study was the relatively large cross-country pool of participants, which made more advanced analyses possible (e.g. clustering). In this study, we used a fully operational system running in real time via a web-based interface. Testing the actual CDSS platform can lead to more robust findings because the user experience is closer to the real-world conditions.

One significant limitation of the study was the absence of an item measuring trust. Without this, we were unable to assess how HCPs characteristics influence the way respondents apply a trust scale. If trust and perceived usefulness are assumed to be closely related, higher levels of trust may increase clinicians’ willingness to rely on system recommendations and thereby enhance perceived clinical usefulness, whereas low trust may limit perceived usefulness regardless of the system’s objective performance. In addition, perceived usefulness may also reinforce trust through repeated positive experiences. The lack of a dedicated trust measure therefore represents a key limitation of this study and highlights an important direction for future research. Furthermore, findings related to AI-assisted CDSS, including the results of this study, may not be directly transferable to non-predictive or rule-based systems.

## Conclusions

This study investigated the relationship between healthcare professionals’ characteristics and the ratings of usability and technology acceptance when evaluating clinical decision support system with an AI module. We found that professionals who were more open to adopting new technologies or who felt more comfortable with technology evaluated the usefulness of the CDSS higher. Also, a prior experience with predictive technologies like the one evaluated in this study was a moderating factor to the satisfaction of the CDSS under evaluation. The results can inform the design of usability and technology acceptance studies, particularly in selecting participant groups to ensure meaningful and representative assessments.

**Fig. 2:**
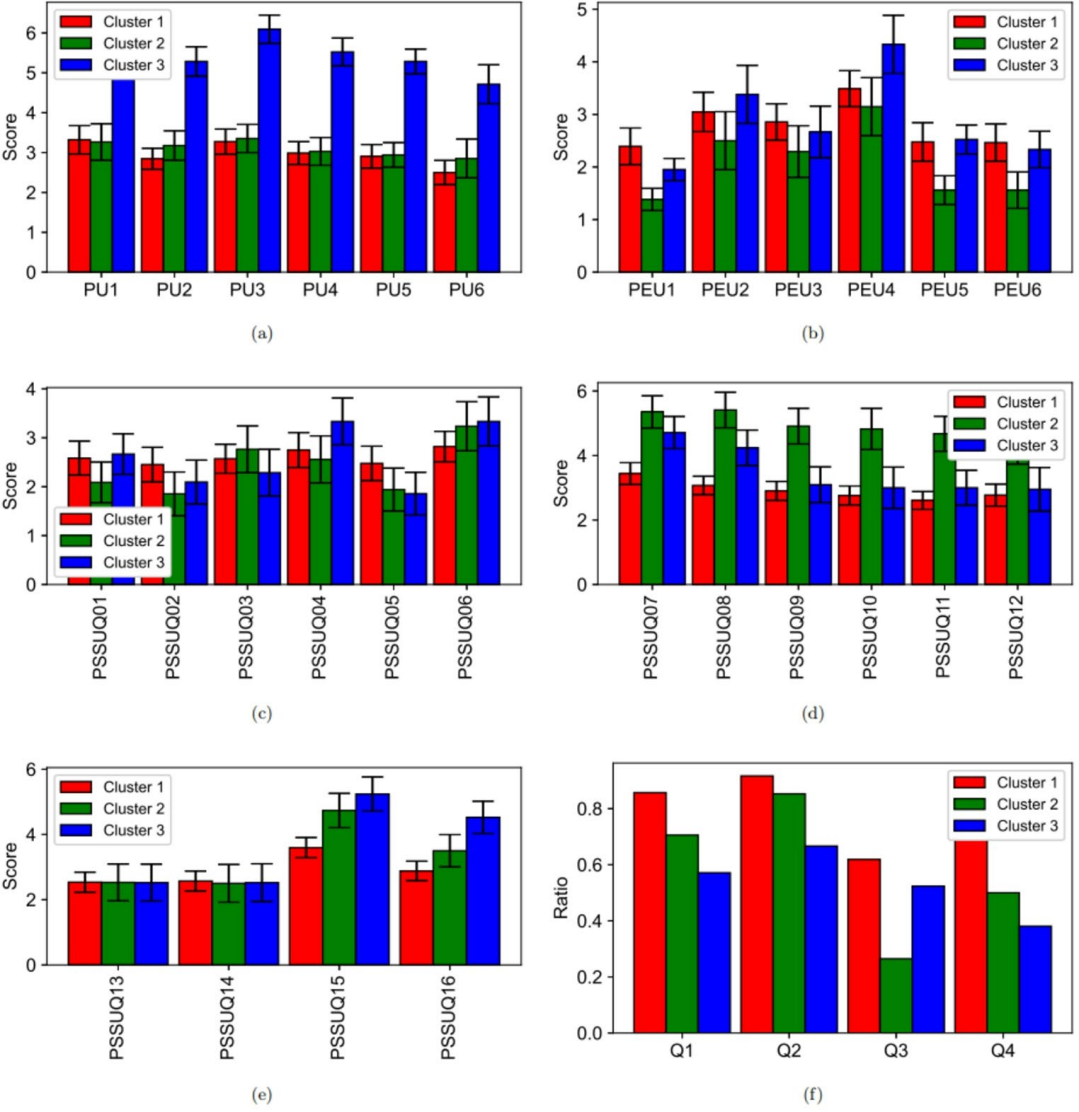
Average responses of (**a**) TAM perceived usefulness, (**b**) TAM perceived ease of use, (**c**) PSSUQ system quality, (**d**) PSSUQ information quality, (**e**) PSSUQ interface quality and (**f**) additional post-questions. Notes: TAM: Technology Acceptance Model; PU: Perceived Usefulness; PEU: Perceived Ease of Use; PSSUQ: Post-Study System Usability Questionnaire; PU1 = Using this tool in my job would enable me to accomplish tasks more quickly, PU2 = Using this tool would improve my job performance, PU3 = Using this tool in my job would increase my productivity, PU4 = Using this tool would enhance my effectiveness on the job, PU5 = Using this tool would make it easier to do my job, PU6 = I would find this tool useful in my job; Scale: 7 = Extremely unlikely, 6 = Quite unlikely, 5 = Slightly unlikely, 4 = Neither, 3 = Slightly likely, 2 = Quite likely, 1 = Extremely likely; PEU1 = Learning to operate this tool would be easy for me, PEU2 = I would find it easy to get this tool to do what I want it to do, PEU3 = My interaction with this tool would be clear and understandable, PEU4 = I would find this tool to be flexible to interact with, PEU5 = It would be easy for me to become skillful at using this tool, PEU6 = I would find this tool easy to use, PEU1 = Learning to operate this tool would be easy for me; Scale: 7 = Extremely unlikely, 6 = Quite unlikely, 5 = Slightly unlikely, 4 = Neither, 3 = Slightly likely, 2 = Quite likely, 1 = Extremely likely PSSUQ1 = Overall, I am satisfied with how easy it is to use this tool, PSSUQ2 = It was simple to use this tool, PSSUQ3 = I was able to complete the tasks and scenarios quickly using this tool, PSSUQ4 = I felt comfortable using this tool, PSSUQ5 = It was easy to learn to use this tool, PSSUQ6 = I believe I could become productive quickly using this tool, PSSUQ7 = The system gave error messages that clearly told me how to fix problems., PSSUQ8 = Whenever I made a mistake using the tool, I could recover easily and quickly., PSSUQ9 = The information (such as online help, on-screen messages, and other documentation provided with this tool was clear., PSSUQ10 = It was easy to find the information I needed., PSSUQ11 = The information was effective in helping me complete the tasks & scenarios., PSSUQ12 = The organization of information on the tool; Scale: 1 (Strongly agree), 2, 3, 4, 5, 6, 7 (Strongly disagree). Q1 = Could the tool give you any valuable information for the care path, Q2 = Could the tool support and guide your work, Q3 = Would the tool raise any legal or ethical considerations if it would be in clinical use, Q4 = I would recommend this tool to colleagues

The members of C1 (who had less prior experience with DSS or prediction tools) felt that the tool had all the functions and capabilities they expected it to have (PSSUQ15) and were more satisfied with the tool (PSSUQ16) than the members of other clusters (*p* < 0.001). Members of C1 rated the tool better than those of C2 and C3, also in the extra questions Q1-Q4 (Fig. 2f). More members from C1 than other clusters evaluated that the tool gives valuable information, supports and guides work, and would recommend the tool to colleagues (*p* < 0.05).

Members of cluster C2 (who were more comfortable with using technology and open to adopting new technologies and had used DSS and prediction tools earlier) perceived the use of the iCARE tool easier (TAM PEU scale) than the other clusters (Fig. 2b). The differences were significant for the responses to the questions: “Learning to operate this tool would be easy for me” (PEU1), “It would be easy for me to become skillful at using this tool” (PEU5), and “I would find this tool easy to use” (PEU6) (*p* < 0.01). The average values were 1.38–1.58, corresponding to the response “extremely or quite likely”. Furthermore, members of C2 evaluated the information quality of the iCARE tool to be significantly worse than the other clusters (Fig. 2d). The average values of the PSSUQ information quality questions 7–12 were higher than in other clusters (*p* < 0.001). The average values were 4.41–5.41 corresponding to a response close to the end of the scale (7 = Strongly disagree). The PSSUQ information quality questions 7–12 focused on how well the tool communicated issues, provided helpful guidance, and enabled users to recover from mistakes efficiently.

Members of cluster C3 (who were less comfortable with using technology) rated the usefulness (TAM PU scale) of the tool worse than those of other clusters (Fig. 2a). The average values of the TAM questions PU1-PU6 for C3 were significantly higher (worse) than for other clusters (*p* < 0.01) (Table S9). The average values of the questions were 4.71–6.1, corresponding to the responses of “slightly or quite unlikely” for the questions on the iCARE tool’s impact on task efficiency, job performance, productivity, and effectiveness. The average values for clusters C1 and C2 (that are more comfortable with using technology) for the same questions were 2.5–3.35, corresponding to the response of “slightly likely”. Members of C3 rated the tool worse than members of C1 and C2, also for the extra questions Q1, Q2 and Q4 (Fig. 2f). Fewer members from cluster C3 than from other clusters answered that the tool gave valuable information for the care path (*p* < 0.05), that the tool supported and guided work (*p* < 0.05) or that they would recommend tool to colleagues (*p* < 0.01).

Supplementary Table S10 presents results for post-hoc interaction analyses of “open to adopting new technology” and “have used prediction tools earlier” on the outcomes of TAM PEU and PSSUQ information quality subscales. Interaction effect of this post-hoc analysis was significant for the outcome of information quality (*p* = 0.03), indicating that prior experience with prediction tools moderates the relationship between openness to adopting new technology and perceived information quality.

Supplementary Table S11 presents the results of post-hoc analyses examining the effect of clinical experience using items of “years in clinical practice” and “years of experience with older adults” on the additional questions measuring the clinical value of the tool. While more years in clinical practice was associated with lower endorsement of one clinical value question (Could the tool give you any valuable information for the care path), no significant associations were observed for the remaining questions. Overall, the results suggested that clinical experience is not consistently associated with perceived clinical value across the assessed questions, and the observed effect appeared to be item specific.

## Supplementary Information

Below is the link to the electronic supplementary material.

Supplementary Material 1

## Data Availability

No datasets were generated or analysed during the current study.
