# Supplementary material for: The Impact of Healthcare Professionals’ Characteristics on the Evaluation of Clinical Decision Support Systems: Insights from a Cross-Country Usability and Technology Acceptance Study of the iCARE Tool
Source: J Med Syst. 2026 Mar 17;50(1):34. doi: 10.1007/s10916-026-02358-5 (PMC12996014; doi:10.1007/s10916-026-02358-5)
Supplement: Supplementary file 1 — Supplementary Material 1 [file 10916_2026_2358_MOESM1_ESM.pdf]

SUPPLEMENTARY FILE: The impact of healthcare professionals' characteristics on the evaluation of clinical decision support systems: insights from a cross-country usability and technology acceptance study of the iCARE tool

Table S1. Pre-questions before the test: Background questions

Question	Options
Age	
Gender	M / F / NA
Current position/job	
Clinical specialty (if applicable)	
Role	
Education/Degree	
Workplace (nursing home / home care)	
Number of years in clinical practice	
Number of years of experience with older adults with complex chronic conditions	
Any experience with interRAI tools?	A) Advanced level of proficiency<br>B) Some working experience<br>C) I have heard about interrail<br>D) No experience
Experience with use of RAI tools, years	

Table S2. Pre-questions before the test: Attitudes towards new technology

How comfortable are you with using technology in general in your daily professional activities?	1 Not comfortable at all<br>2<br>3<br>4<br>5 Very comfortable
How open are you to adopting new technologies in general in your healthcare practice?	1Not open at all<br>2<br>3<br>4<br>5 Very open
What kind of clinical decision support tool would be beneficial for you in patient care? (Briefly describe)	
Have you used any decision support systems in your work?	No<br>Yes
If yes, please state what kind of tool	
Have you used any decision support systems with predictions for patient trajectories?	No<br>Yes
If yes, please state what kind of tool	

Table S3. Post-questions after the test: Technology Acceptance Model (TAM).
Rating scale: 7 = Extremely unlikely, 6 = Quite unlikely, 5 = Slightly unlikely, 4 = Neither, 3 = Slightly likely, 2 = Quite likely, 1 = Extremely likely

Perceived Usefulness (PU)
1. Using this tool in my job would enable me to accomplish tasks more quickly.
2. Using this tool would improve my job performance.
3. Using this tool in my job would increase my productivity.
4. Using this tool would enhance my effectiveness on the job.
5. Using this tool would make it easier to do my job.
6. I would find this tool useful in my job.
Perceived Ease of Use (PEU)
7. Learning to operate this tool would be easy for me.
8. I would find it easy to get this tool to do what I want it to do.
9. My interaction with this tool would be clear and understandable.
10. I would find this tool to be flexible to interact with.
11. It would be easy for me to become skillful at using this tool.
12. I would find this tool easy to use.

Table S4. Post-questions after the test: Post-Study System Usability Questionnaire (PSSUQ).
Rating scale: 1 (Strongly agree), 2, 3, 4, 5, 6, 7 (Strongly disagree)

1. Overall, I am satisfied with how easy it is to use this tool.
2. It was simple to use this tool.
3. I was able to complete the tasks and scenarios quickly using this tool.
4. I felt comfortable using this tool.
5. It was easy to learn to use this tool.
6. I believe I could become productive quickly using this tool.
7. The system gave error messages that clearly told me how to fix problems.
8. Whenever I made a mistake using the tool, I could recover easily and quickly.
9. The information (such as online help, on-screen messages, and other documentation provided with this tool was clear.
10. It was easy to find the information I needed.
11. The information was effective in helping me complete the tasks & scenarios.
12. The organization of information on the tool screens was clear.
13. The interface of this tool was pleasant.
14. I liked using the interface of this tool.
15. This tool has all the functions and capabilities I expect it to have.
16. Overall, I am satisfied with this tool.

Table S5. Post-questions after the test: Additional questions

1. Could the tool give you any valuable information for the care path?	NO / YES
2. Could the tool support and guide your work?	NO / YES
3. Would the tool raise any legal or ethical considerations if it would be in clinical use?	NO / YES
4. I would recommend this tool to colleagues?	NO / YES

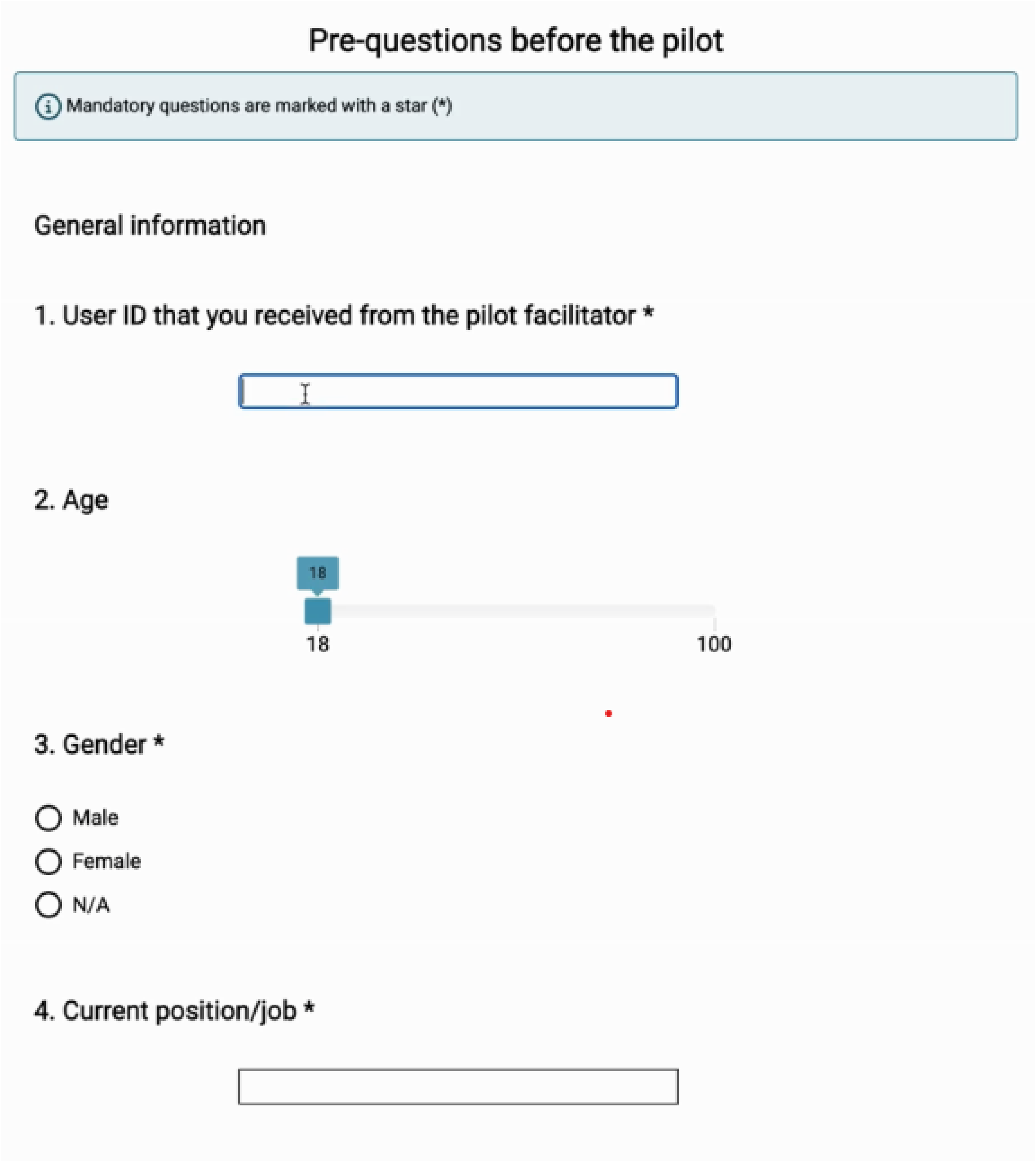

Figure S1. For each participant, first a pre-questionnaire (Table S1) was filled out, which consisted of background questions (socio-demographic information and questions about education, profession, actual position and professional experience), as well as questions related to attitudes towards new technology (Table S2). This figure presents only part of the questions.

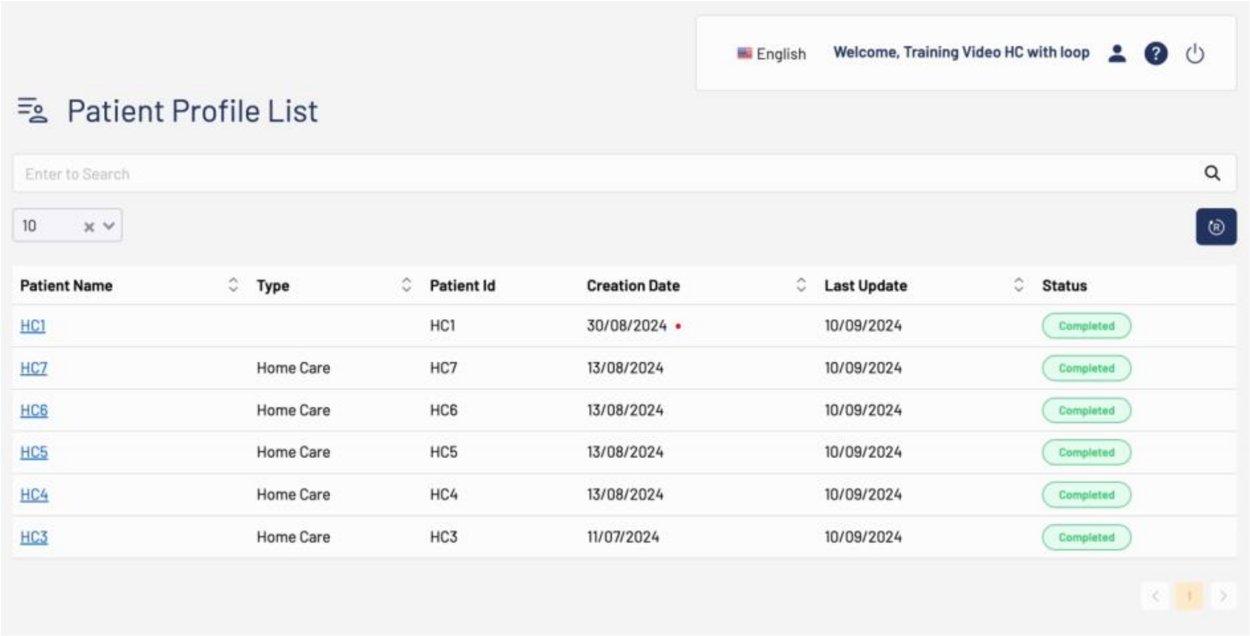

Figure S2. After filling out the pre-questionnaire (Figure S1; Tables S1 and S2), participants selected one patient case at time for evaluation.

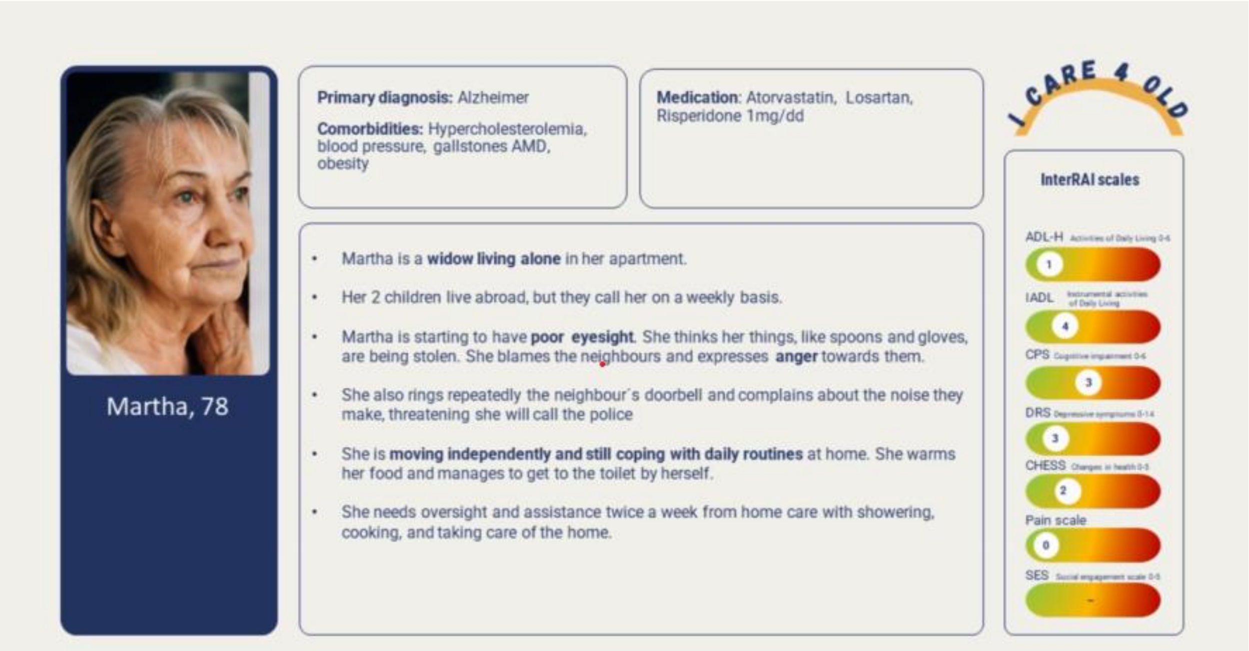

Figure S3. Summary information of one patient case

Notes: The patient cases were hypothetical home or long-term facility clients created by the experts of I-CARE4OLD project. For each patient case, the experts defined summary information and interRAI assessment values. Based on this information, the iCARE tool calculated risk predictions and treatment effect estimates.

Predictions in 12 months	Hospitalization	Function worsening (ADL + IADL)	Cognition worsening (CPS)	Health Instability (CHESS)	Other (Falls)	Quality of Life worsening (HUI3)
Risk of decline / of event	15.14%	62.61%				8.68%
What if you decide to:	Predicted change					
Stop Antipsychotics	-1.159%	-1.02%	4.43%	-9.71%		
Start Anticholinergics	8.784%	8.68%	3.25%		7.002%	
Start Physiotherapy			Predicted ITE Standard Model: [0.] Predicted percentage [0.]			

Figure S4. Risk predictions and treatment effect estimations for hospitalization, functional and cognitive worsening, health instability (CHESS), falls and quality of life worsening calculated by iCARE tool for one patient.

Notes: ADL: Activity of Daily Life, IADL: Instrumental Activity of Daily Life; CHESS: Changes in Health, End-stage disease and Symptoms and Signs; HUI3: Health Utility Index Mark 3

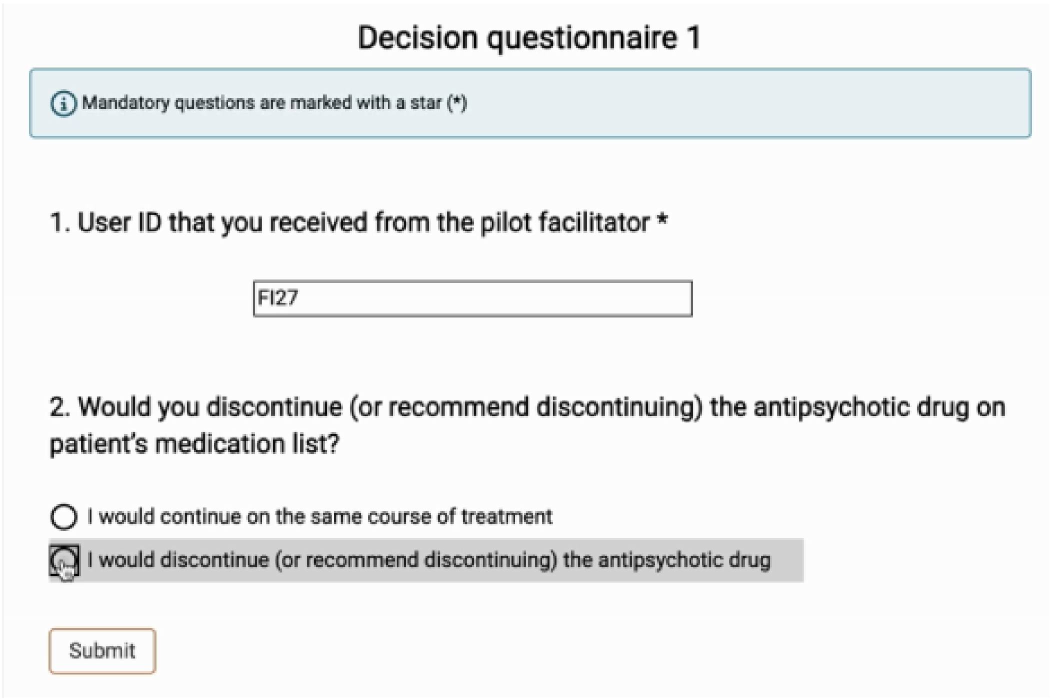

(a)

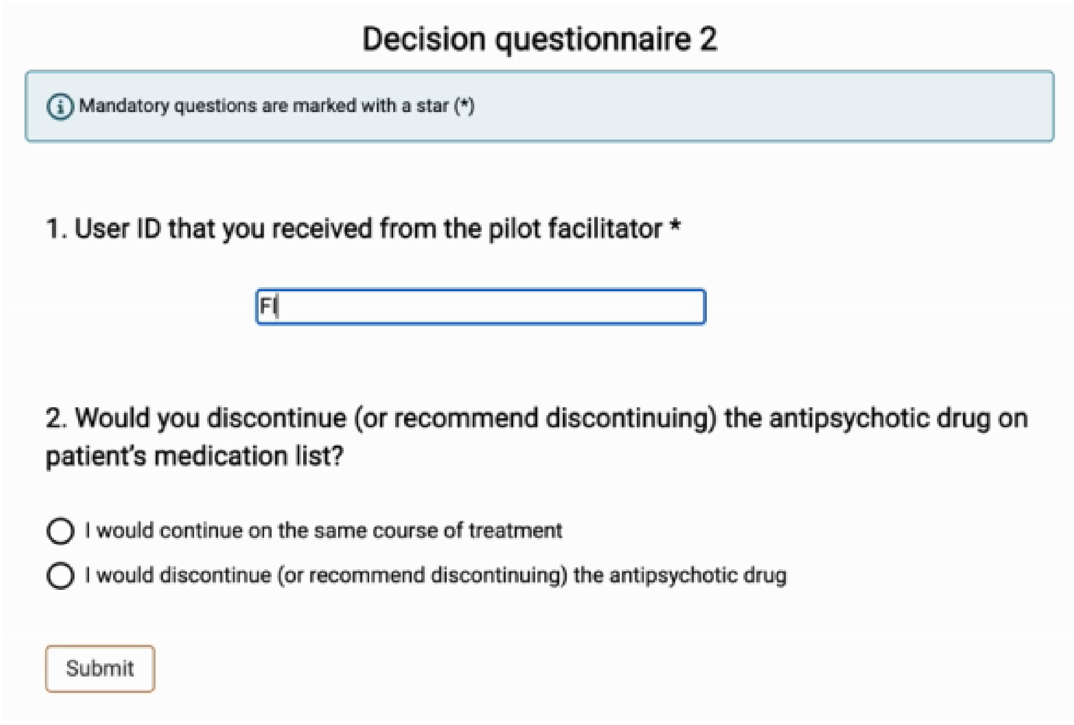

(b)

Figure S5. One task of the participants was to make treatment decision before (a) and update the decision (b) after they reviewed the predictions (Figure S4).

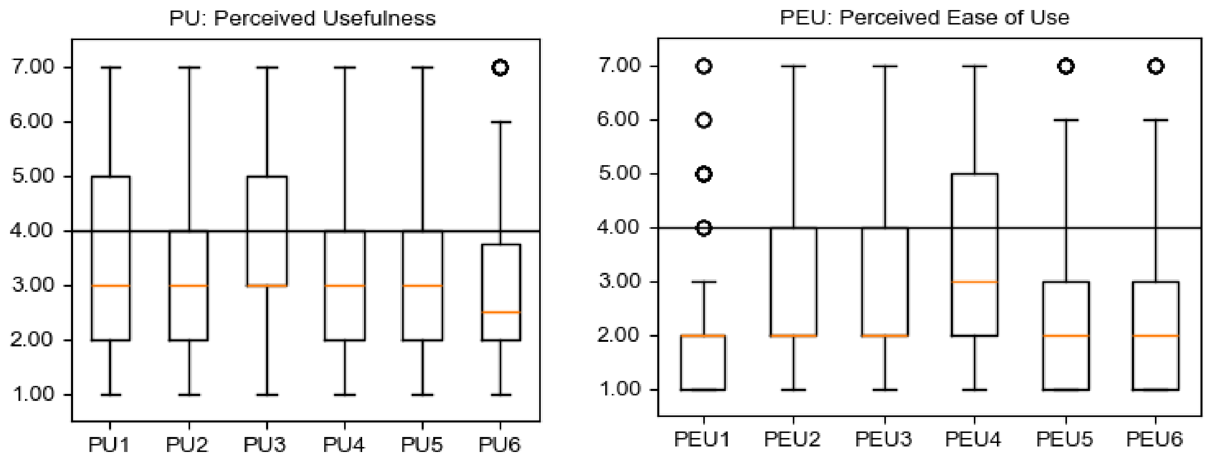

(a) (b)

Figure S6. Technology Acceptance Model (TAM): (a) Perceived Usefulness (PU) and (b) Perceived Ease of Use (PEU)

Notes:

Scale: 7 = Extremely unlikely, 6 = Quite unlikely, 5 = Slightly unlikely, 4 = Neither, 3 = Slightly likely, 2 = Quite likely, 1 = Extremely likely

PU1 = Using this tool in my job would enable me to accomplish tasks more quickly.

PU2 = Using this tool would improve my job performance.

PU3 = Using this tool in my job would increase my productivity.

PU4 = Using this tool would enhance my effectiveness on the job.

PU5 = Using this tool would make it easier to do my job.

PU6 = I would find this tool useful in my job.

PEU1 = Learning to operate this tool would be easy for me.

PEU2 = I would find it easy to get this tool to do what I want it to do.

PEU3 = My interaction with this tool would be clear and understandable.

PEU4 = I would find this tool to be flexible to interact with.

PEU5 = It would be easy for me to become skillful at using this tool.

PEU6 = I would find this tool easy to use.

Values were measured on an ordinal Likert scale. The median of 2.5 for PU6 reflects an even number of observations, with the two central responses being 2 and 3, resulting in a midpoint median rather than an actual response option.

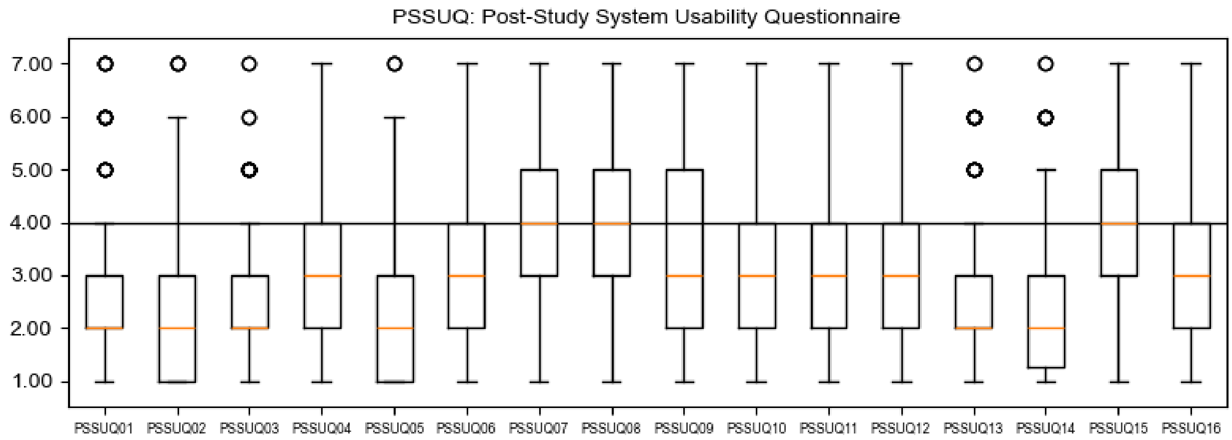

Figure S7. Post-Study System Usability Questionnaire (PSSUQ)

Notes:

Scale: 1 (Strongly agree), 2, 3, 4, 5, 6, 7 (Strongly disagree)

PSSUQ1 = Overall, I am satisfied with how easy it is to use this tool.
PSSUQ2 = It was simple to use this tool.
PSSUQ3 = I was able to complete the tasks and scenarios quickly using this tool.
PSSUQ4 = I felt comfortable using this tool.
PSSUQ5 = It was easy to learn to use this tool.
PSSUQ6 = I believe I could become productive quickly using this tool.
PSSUQ7 = The system gave error messages that clearly told me how to fix problems.
PSSUQ8 = Whenever I made a mistake using the tool, I could recover easily and quickly.
PSSUQ9 = The information (such as online help, on-screen messages, and other documentation provided with this tool was clear.
PSSUQ10 = It was easy to find the information I needed.
PSSUQ11 = The information was effective in helping me complete the tasks & scenarios.
PSSUQ12 = The organization of information on the tool screens was clear.
PSSUQ13 = The interface of this tool was pleasant.
PSSUQ14 = I liked using the interface of this tool.
PSSUQ15 = This tool has all the functions and capabilities I expect it to have.
PSSUQ16 = Overall, I am satisfied with this tool.

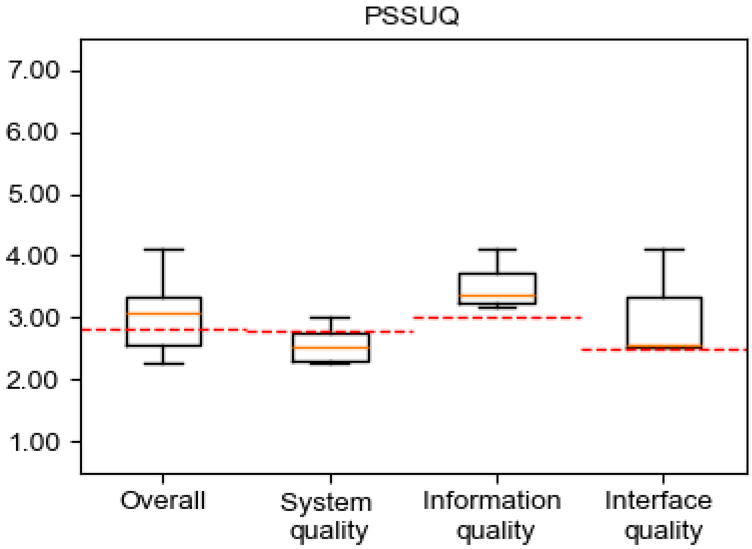

Figure S8. Post-Study System Usability Questionnaire (PSSUQ) mean values of overall and the factors of system, information and interface quality. The benchmarking scores are marked red lines*.

Notes:

- Overall: Average the responses for Items 1 through 16 (all the items)
- System quality: Average Items 1 through 6
- Information quality: Average Items 7 through 12
- Interface quality: Average Items 13 through 15

*Sauro, J.; Lewis, J.R. Quantifying the User Experience: Practical Statistics for User Research. Morgan Kaufmann; Elsevier: Amsterdam, The Netherlands, 2016

Table S6. Hypothesis tests. Model H1: Regression models of openness to adopting new technology" **. Model H2: Regression models of comfortable with technology***. The models were adjusted by age, gender, education and years with use of RAI tools.

Question	Modell H1 Parameter	95% CI	p-values	Model H2 Parameter	95% CI	p-values
PU1 = Using this tool in my job would enable me to accomplish tasks more quickly.	-0.744	[-1.037, -0.452]	**0.0***	-0.442	[-0.713, -0.17]	**0.002***
PU2 = Using this tool would improve my job performance.	-0.551	[-0.805, -0.297]	**0.0***	-0.332	[-0.565, -0.1]	0.005
PU3 = Using this tool in my job would increase my productivity.	-0.641	[-0.912, -0.369]	**0.0***	-0.411	[-0.663, -0.159]	**0.002***
PU4 = Using this tool would enhance my effectiveness on the job.	-0.553	[-0.817, -0.289]	**0.0***	-0.349	[-0.589, -0.11]	0.005
PU5 = Using this tool would make it easier to do my job.	-0.533	[-0.803, -0.263]	**0.0***	-0.315	[-0.562, -0.068]	0.013
PU6 = I would find this tool useful in my job.	-0.66	[-0.939, -0.381]	**0.0***	-0.413	[-0.67, -0.155]	**0.002***
PEU1 = Learning to operate this tool would be easy for me.	-0.49	[-0.732, -0.248]	**0.0***	-0.43	[-0.65, -0.21]	**0.0***
PEU2 = I would find it easy to get this tool to do what I want it to do.	-0.832	[-1.12, -0.544]	**0.0***	-0.447	[-0.72, -0.174]	**0.002***
PEU3 = My interaction with this tool would be clear and understandable.	-0.647	[-0.903, -0.39]	**0.0***	-0.453	[-0.693, -0.214]	**0.0***
PEU4 = I would find this tool to be flexible to interact with.	-0.44	[-0.723, -0.157]	**0.003***	-0.182	[-0.441, 0.076]	0.165
PEU5 = It would be easy for me to become skillful at using this tool.	-0.575	[-0.831, -0.32]	**0.0***	-0.417	[-0.658, -0.176]	**0.001***
PEU6 = I would find this tool easy to use.	-0.402	[-0.667, -0.137]	**0.003***	-0.226	[-0.466, 0.015]	0.066
PSSUQ1 = Overall, I am satisfied with how easy it is to use this tool.	-0.394	[-0.653, -0.134]	**0.003***	-0.37	[-0.602, -0.138]	**0.002***
PSSUQ2 = It was simple to use this tool.	-0.318	[-0.577, -0.058]	0.017	-0.303	[-0.534, -0.072]	0.011
PSSUQ3 = I was able to complete the tasks and scenarios quickly using this tool.	-0.273	[-0.502, -0.043]	0.02	-0.142	[-0.351, 0.066]	0.179
PSSUQ4 = I felt comfortable using this tool.	-0.628	[-0.897, -0.358]	**0.0***	-0.426	[-0.674, -0.178]	**0.001***
PSSUQ5 = It was easy to learn to use this tool.	-0.295	[-0.548, -0.042]	0.022	-0.166	[-0.396, 0.064]	0.156
PSSUQ6 = I believe I could become productive quickly using this tool.	-0.417	[-0.688, -0.146]	**0.003***	-0.264	[-0.51, -0.017]	0.036
PSSUQ7 = The system gave error messages that clearly told me how to fix problems.	-0.613	[-0.916, -0.31]	**0.0***	-0.11	[-0.394, 0.175]	0.447
PSSUQ8 = Whenever I made a mistake using the tool, I could recover easily and quickly.	-0.263	[-0.573, 0.046]	0.095	0.006	[-0.27, 0.283]	0.965
PSSUQ9 = The information (such as online help, on-screen messages, and other documentation provided with this tool was clear.	-0.384	[-0.682, -0.086]	0.012	0.028	[-0.243, 0.299]	0.837
PSSUQ10 = It was easy to find the information I needed.	-0.339	[-0.636, -0.042]	0.026	-0.129	[-0.395, 0.137]	0.34
PSSUQ11 = The information was effective in helping me complete the tasks & scenarios.	-0.279	[-0.568, 0.011]	0.059	-0.074	[-0.334, 0.186]	0.576
PSSUQ12 = The organization of information on the tool screens was clear.	-0.054	[-0.367, 0.258]	0.732	0.146	[-0.13, 0.422]	0.298
PSSUQ13 = The interface of this tool was pleasant.	-0.446	[-0.693, -0.199]	**0.0***	-0.131	[-0.36, 0.098]	0.259
PSSUQ14 = I liked using the interface of this tool.	-0.583	[-0.825, -0.341]	**0.0***	-0.224	[-0.455, 0.007]	0.057
PSSUQ15 = This tool has all the functions and capabilities I expect it to have.	-0.466	[-0.74, -0.192]	**0.001***	-0.214	[-0.464, 0.037]	0.094
PSSUQ16 = Overall, I am satisfied with this tool.	-0.466	[-0.723, -0.209]	**0.0***	-0.342	[-0.573, -0.11]	0.004
Could the tool give you any valuable information for the care path?	0.005	[-0.431, 0.442]	0.981	-0.012	[-0.395, 0.37]	0.949
Could the tool support and guide your work?	0.468	[0.006, 0.931]	0.047	0.446	[0.024, 0.867]	0.038
Would the tool raise any legal or ethical considerations if it would be in clinical use?	0.008	[-0.349, 0.365]	0.965	0.013	[-0.303, 0.33]	0.934
I would recommend this tool to colleagues?	0.488	[0.102, 0.874]	0.013	0.247	[-0.087, 0.582]	0.147

Notes: *Significance level of Holm-Bonferroni-adjusted p-value <0.05; ** How open are you to adopting new technologies in general in your healthcare practice? 1 (Not open at all), 2, 3, 4, 5 (Very open); *** How comfortable are you with using technology in general in your daily professional activities? 1 (Not comfortable at all), 2, 3, 4, 5 (Very comfortable); PU: Perceived Usefulness; PEU: Perceived Ease of Use; PSSUQ: Post-Study System Usability Questionnaire; CI: Confidence Intervals

Table S7. Hypothesis tests. Model H3: Regression models of younger age. Model H4: Regression models of a higher educations. The model H3 was adjusted by gender, education and years with use of RAI tools and H4 age, gender and years with use of RAI tools.

Question	Model H3 Pars	95% CI	p-values	Model H4 Pars	95% CI	p-values
PU1 = Using this tool in my job would enable me to accomplish tasks more quickly.	-0.003	[-0.03, 0.024]	0.837	0.162	[-0.61, 0.934]	0.679
PU2 = Using this tool would improve my job performance.	0.009	[-0.014, 0.032]	0.447	-0.018	[-0.672, 0.636]	0.957
PU3 = Using this tool in my job would increase my productivity.	0.004	[-0.02, 0.029]	0.727	0.465	[-0.243, 1.174]	0.196
PU4 = Using this tool would enhance my effectiveness on the job.	0.005	[-0.018, 0.029]	0.648	-0.11	[-0.787, 0.567]	0.749
PU5 = Using this tool would make it easier to do my job.	0.01	[-0.014, 0.034]	0.401	-0.121	[-0.81, 0.568]	0.729
PU6 = I would find this tool useful in my job.	0.017	[-0.009, 0.042]	0.199	0.013	[-0.715, 0.74]	0.973
PEU1 = Learning to operate this tool would be easy for me.	0.016	[-0.005, 0.038]	0.132	-0.752	[-1.371, -0.133]	0.018
PEU2 = I would find it easy to get this tool to do what I want it to do.	0.002	[-0.025, 0.029]	0.867	-0.122	[-0.898, 0.654]	0.756
PEU3 = My interaction with this tool would be clear and understandable.	0.012	[-0.011, 0.036]	0.294	-0.724	[-1.398, -0.049]	0.036
PEU4 = I would find this tool to be flexible to interact with.	0.014	[-0.011, 0.039]	0.265	0.348	[-0.361, 1.056]	0.334
PEU5 = It would be easy for me to become skillful at using this tool.	0.021	[-0.002, 0.044]	0.072	-1.037	[-1.699, -0.375]	0.002
PEU6 = I would find this tool easy to use.	0.01	[-0.013, 0.033]	0.402	-0.363	[-1.024, 0.299]	0.28
PSSUQ1 = Overall, I am satisfied with how easy it is to use this tool.	0.022	[-0.001, 0.045]	0.056	-0.661	[-1.31, -0.012]	0.046
PSSUQ2 = It was simple to use this tool.	0.013	[-0.009, 0.035]	0.254	-0.512	[-1.152, 0.128]	0.116
PSSUQ3 = I was able to complete the tasks and scenarios quickly using this tool.	-0.003	[-0.023, 0.017]	0.756	-0.482	[-1.048, 0.084]	0.094
PSSUQ4 = I felt comfortable using this tool.	0.002	[-0.022, 0.026]	0.877	-0.428	[-1.129, 0.273]	0.229
PSSUQ5 = It was easy to learn to use this tool.	0.018	[-0.004, 0.04]	0.103	-0.591	[-1.214, 0.033]	0.063
PSSUQ6 = I believe I could become productive quickly using this tool.	0.013	[-0.011, 0.037]	0.276	-0.556	[-1.233, 0.121]	0.107
PSSUQ7 = The system gave error messages that clearly told me how to fix problems.	0.009	[-0.018, 0.036]	0.509	-0.241	[-1.016, 0.534]	0.54
PSSUQ8 = Whenever I made a mistake using the tool, I could recover easily and quickly.	0.012	[-0.014, 0.038]	0.366	0.14	[-0.616, 0.896]	0.716
PSSUQ9 = The information (such as online help, on-screen messages, and other documentation provided with this tool was clear.	0.001	[-0.024, 0.027]	0.91	-0.066	[-0.803, 0.672]	0.86
PSSUQ10 = It was easy to find the information I needed.	-0.005	[-0.031, 0.02]	0.688	0.28	[-0.452, 1.012]	0.451
PSSUQ11 = The information was effective in helping me complete the tasks & scenarios.	0.004	[-0.02, 0.029]	0.736	-0.224	[-0.934, 0.485]	0.533
PSSUQ12 = The organization of information on the tool screens was clear.	0.012	[-0.015, 0.038]	0.384	0.079	[-0.678, 0.835]	0.837
PSSUQ13 = The interface of this tool was pleasant.	0.009	[-0.013, 0.031]	0.41	-0.327	[-0.952, 0.298]	0.303
PSSUQ14 = I liked using the interface of this tool.	0.008	[-0.014, 0.03]	0.447	-0.479	[-1.113, 0.154]	0.137
PSSUQ15 = This tool has all the functions and capabilities I expect it to have.	0.015	[-0.009, 0.039]	0.226	0.137	[-0.552, 0.827]	0.695
PSSUQ16 = Overall, I am satisfied with this tool.	0.01	[-0.013, 0.032]	0.392	-0.1	[-0.751, 0.551]	0.761
Could the tool give you any valuable information for the care path?	-0.037	[-0.074, 0.0]	0.051	-0.948	[-2.277, 0.381]	0.162
Could the tool support and guide your work?	-0.014	[-0.057, 0.03]	0.54	0.651	[-0.59, 1.892]	0.304
Would the tool raise any legal or ethical considerations if it would be in clinical use?	-0.013	[-0.043, 0.018]	0.413	0.114	[-0.75, 0.979]	0.796
I would recommend this tool to colleagues?	-0.003	[-0.035, 0.029]	0.853	-0.59	[-1.617, 0.436]	0.26

Notes: *Significance level of Holm-Bonferroni-adjusted p-value <0.05; ** How open are you to adopting new technologies in general in your healthcare practice? 1 (Not open at all), 2, 3, 4, 5 (Very open); *** How comfortable are you with using technology in general in your daily professional activities? 1 (Not comfortable at all), 2, 3, 4, 5 (Very comfortable); PU: Perceived Usefulness; PEU: Perceived Ease of Use; PSSUQ: Post-Study System Usability Questionnaire; CI: Confidence Intervals

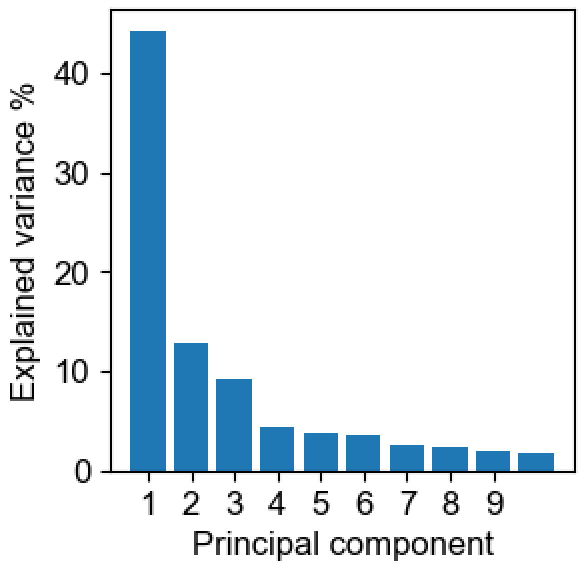

Figure S9. Eigenvalues for the 10 first principal components of the data set. The principal components are sorted in decreasing order of the explained variance.

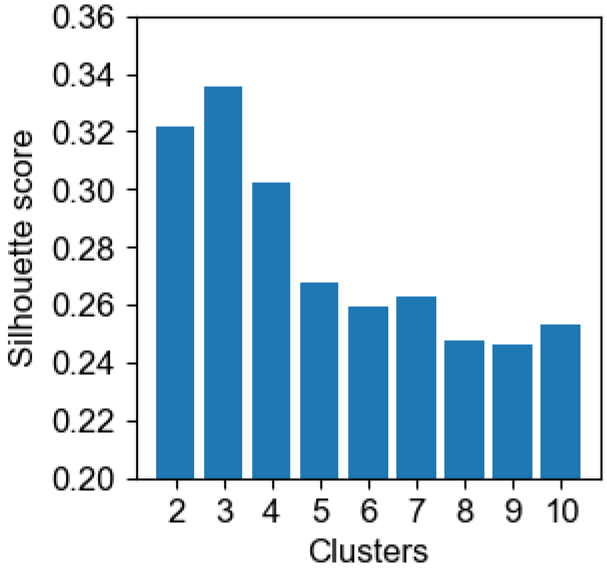

Figure S10. Silhouette score for the clustering solutions from 2 to 10 clusters.

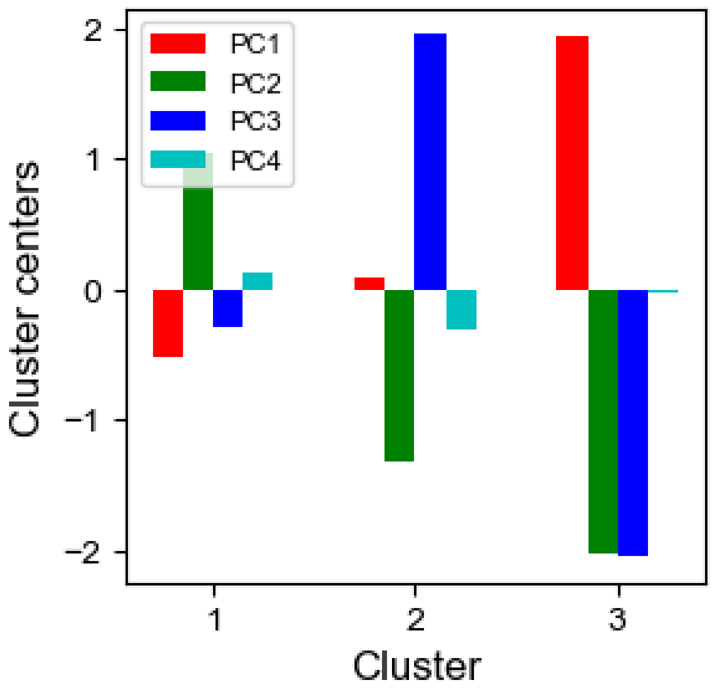

Figure S11. Cluster centers in the space of the 4 principal components derived from TAM (Technology Acceptance Model) and PSSUQ (Post-Study System Usability) questionnaires.

Table S8. Loadings of the principal components 1-4

Variables (General system quality)	PC1 loadings
PSSUQ16 = Overall, I am satisfied with this tool.	0.8026
PSSUQ4 = I felt comfortable using this tool.	0.7998
PSSUQ1 = Overall, I am satisfied with how easy it is to use this tool.	0.7785
PSSUQ2 = It was simple to use this tool.	0.7574
PEU3 = My interaction with this tool would be clear and understandable.	0.7505
PSSUQ5 = It was easy to learn to use this tool.	0.7447
PEU5 = It would be easy for me to become skillful at using this tool.	0.7411
PEU6 = I would find this tool easy to use.	0.7161
PU2 = Using this tool would improve my job performance.	0.7159
PU6 = I would find this tool useful in my job.	0.7055
PEU1 = Learning to operate this tool would be easy for me.	0.7049
PSSUQ6 = I believe I could become productive quickly using this tool.	0.6936
PU5 = Using this tool would make it easier to do my job.	0.6898
PSSUQ13 = The interface of this tool was pleasant.	0.6887
PSSUQ14 = I liked using the interface of this tool.	0.6873
PSSUQ3 = I was able to complete the tasks and scenarios quickly using this tool.	0.6826
PU4 = Using this tool would enhance my effectiveness on the job.	0.6810
PEU2 = I would find it easy to get this tool to do what I want it to do.	0.6757
PU3 = Using this tool in my job would increase my productivity.	0.6603
PU1 = Using this tool in my job would enable me to accomplish tasks more quickly.	0.6513
PSSUQ11 = The information was effective in helping me complete the tasks & scenarios.	0.5811
PSSUQ10 = It was easy to find the information I needed.	0.5523
PSSUQ9 = The information (such as online help, on-screen messages, and other documentation provided with this tool was clear.	0.5443
PSSUQ7 = The system gave error messages that clearly told me how to fix problems.	0.5242
PSSUQ12 = The organization of information on the tool screens was clear.	0.5239
PSSUQ15 = This tool has all the functions and capabilities I expect it to have.	0.5079
PEU4 = I would find this tool to be flexible to interact with.	0.5051
PSSUQ8 = Whenever I made a mistake using the tool, I could recover easily and quickly.	0.4321

Variables (High Perceived Ease of Use and Low Perceived utility)	PC2 loadings
PEU1 = Learning to operate this tool would be easy for me.	0.5535
PSSUQ15 = This tool has all the functions and capabilities I expect it to have.	-0.5340
PSSUQ2 = It was simple to use this tool.	0.5266
PEU6 = I would find this tool easy to use.	0.5224
PSSUQ8 = Whenever I made a mistake using the tool, I could recover easily and quickly.	-0.5183
PSSUQ5 = It was easy to learn to use this tool.	0.5174
PU3 = Using this tool in my job would increase my productivity.	-0.4560
PSSUQ1 = Overall, I am satisfied with how easy it is to use this tool.	0.4383

PSSUQ7 = The system gave error messages that clearly told me how to fix problems.	-0.4149
PU2 = Using this tool would improve my job performance.	-0.3991
PU4 = Using this tool would enhance my effectiveness on the job.	-0.3883
PEU5 = It would be easy for me to become skillful at using this tool.	0.3803
PU1 = Using this tool in my job would enable me to accomplish tasks more quickly.	-0.3547
PU6 = I would find this tool useful in my job.	-0.3283
PU5 = Using this tool would make it easier to do my job.	-0.3202
PEU3 = My interaction with this tool would be clear and understandable.	0.2915
PSSUQ11 = The information was effective in helping me complete the tasks & scenarios.	-0.2911
PSSUQ16 = Overall, I am satisfied with this tool.	-0.2836
PSSUQ3 = I was able to complete the tasks and scenarios quickly using this tool.	0.2808
PSSUQ10 = It was easy to find the information I needed.	-0.2320
PSSUQ9 = The information (such as online help, on-screen messages, and other documentation provided with this tool was clear.	-0.2231
PSSUQ4 = I felt comfortable using this tool.	0.1950
PSSUQ14 = I liked using the interface of this tool.	0.1909
PSSUQ13 = The interface of this tool was pleasant.	0.1437
PSSUQ12 = The organization of information on the tool screens was clear.	-0.1229
PEU4 = I would find this tool to be flexible to interact with.	-0.1076
PEU2 = I would find it easy to get this tool to do what I want it to do.	0.1038
PSSUQ6 = I believe I could become productive quickly using this tool.	-0.0371

**Variables (Information quality)**	**PC3 loadings**
PSSUQ10 = It was easy to find the information I needed.	0.5731
PSSUQ9 = The information (such as online help, on-screen messages, and other documentation provided with this tool was clear.	0.5397
PSSUQ11 = The information was effective in helping me complete the tasks & scenarios.	0.4826
PSSUQ12 = The organization of information on the tool screens was clear.	0.4802
PU5 = Using this tool would make it easier to do my job.	-0.4258
PSSUQ8 = Whenever I made a mistake using the tool, I could recover easily and quickly.	0.4248
PU4 = Using this tool would enhance my effectiveness on the job.	-0.4085
PU3 = Using this tool in my job would increase my productivity.	-0.3901
PU1 = Using this tool in my job would enable me to accomplish tasks more quickly.	-0.3811
PU2 = Using this tool would improve my job performance.	-0.3478
PU6 = I would find this tool useful in my job.	-0.2971
PSSUQ7 = The system gave error messages that clearly told me how to fix problems.	0.2829
PSSUQ3 = I was able to complete the tasks and scenarios quickly using this tool.	0.2476
PEU5 = It would be easy for me to become skillful at using this tool.	-0.2421
PSSUQ13 = The interface of this tool was pleasant.	0.2408
PEU4 = I would find this tool to be flexible to interact with.	-0.2249
PSSUQ14 = I liked using the interface of this tool.	0.2234
PEU2 = I would find it easy to get this tool to do what I want it to do.	-0.1960
PEU1 = Learning to operate this tool would be easy for me.	-0.1503

PSSUQ5 = It was easy to learn to use this tool.	0.1256
PEU6 = I would find this tool easy to use.	-0.1171
PSSUQ15 = This tool has all the functions and capabilities I expect it to have.	0.0748
PSSUQ2 = It was simple to use this tool.	0.0494
PSSUQ6 = I believe I could become productive quickly using this tool.	0.0405
PEU3 = My interaction with this tool would be clear and understandable.	-0.0336
PSSUQ16 = Overall, I am satisfied with this tool.	0.0226
PSSUQ1 = Overall, I am satisfied with how easy it is to use this tool.	-0.0049
PSSUQ4 = I felt comfortable using this tool.	0.0037

**Variables**	**PC4 loadings**
PEU4 = I would find this tool to be flexible to interact with.	0.4744
PSSUQ6 = I believe I could become productive quickly using this tool.	-0.3999
PSSUQ14 = I liked using the interface of this tool.	0.3936
PSSUQ13 = The interface of this tool was pleasant.	0.3729
PEU2 = I would find it easy to get this tool to do what I want it to do.	0.3185
PU6 = I would find this tool useful in my job.	-0.2749
PSSUQ15 = This tool has all the functions and capabilities I expect it to have.	0.2518
PU2 = Using this tool would improve my job performance.	-0.1921
PU5 = Using this tool would make it easier to do my job.	-0.1894
PSSUQ11 = The information was effective in helping me complete the tasks & scenarios.	-0.1776
PU3 = Using this tool in my job would increase my productivity.	0.1701
PEU1 = Learning to operate this tool would be easy for me.	-0.1674
PSSUQ8 = Whenever I made a mistake using the tool, I could recover easily and quickly.	-0.1660
PSSUQ1 = Overall, I am satisfied with how easy it is to use this tool.	-0.1636
PSSUQ9 = The information (such as online help, on-screen messages, and other documentation provided with this tool was clear.	-0.1568
PSSUQ3 = I was able to complete the tasks and scenarios quickly using this tool.	-0.1293
PSSUQ4 = I felt comfortable using this tool.	0.1202
PSSUQ12 = The organization of information on the tool screens was clear.	0.1190
PU1 = Using this tool in my job would enable me to accomplish tasks more quickly.	0.0844
PEU5 = It would be easy for me to become skillful at using this tool.	-0.0799
PSSUQ2 = It was simple to use this tool.	-0.0778
PSSUQ16 = Overall, I am satisfied with this tool.	0.0658
PU4 = Using this tool would enhance my effectiveness on the job.	-0.0643
PEU3 = My interaction with this tool would be clear and understandable.	0.0640
PEU6 = I would find this tool easy to use.	-0.0574
PSSUQ7 = The system gave error messages that clearly told me how to fix problems.	0.0301
PSSUQ10 = It was easy to find the information I needed.	-0.0129
PSSUQ5 = It was easy to learn to use this tool.	-0.0118

Table S9. Mean question answers in different clusters.

Item name	All	C1	C2	C3	P-value (C1 vrs. rest)	P-value (C2 vrs. rest)	P-value (C3 vrs. rest)
PU1 = Using this tool in my job would enable me to accomplish tasks more quickly., mean (std)	3.69 (1.76)	3.32 (1.63)	3.26 (1.31)	5.86 (1.31)	0.002	0.222	<0.001
PU2 = Using this tool would improve my job performance., mean (std)	3.29 (1.49)	2.85 (1.21)	3.18 (1.06)	5.29 (1.55)	<0.001	0.728	<0.001
PU3 = Using this tool in my job would increase my productivity., mean (std)	3.72 (1.63)	3.27 (1.45)	3.35 (1.01)	6.1 (0.94)	<0.001	0.247	<0.001
PU4 = Using this tool would enhance my effectiveness on the job., mean (std)	3.38 (1.54)	2.99 (1.31)	3.03 (1.0)	5.52 (1.4)	<0.001	0.382	<0.001
PU5 = Using this tool would make it easier to do my job., mean (std)	3.27 (1.57)	2.9 (1.37)	2.94 (0.89)	5.29 (1.74)	0.001	0.546	<0.001
PU6 = I would find this tool useful in my job., mean (std)	2.92 (1.68)	2.5 (1.4)	2.85 (1.4)	4.71 (1.98)	<0.001	0.736	<0.001
PEU1 = Learning to operate this tool would be easy for me., mean (std)	2.08 (1.45)	2.39 (1.61)	1.38 (0.6)	1.95 (1.4)	<0.001	<0.001	0.430
PEU2 = I would find it easy to get this tool to do what I want it to do., mean (std)	2.96 (1.77)	3.05 (1.72)	2.5 (1.58)	3.38 (2.16)	0.308	0.074	0.453
PEU3 = My interaction with this tool would be clear and understandable., mean (std)	2.69 (1.57)	2.86 (1.59)	2.29 (1.4)	2.67 (1.68)	0.08	0.075	0.804
PEU4 = I would find this tool to be flexible to interact with., mean (std)	3.53 (1.63)	3.49 (1.58)	3.15 (1.58)	4.33 (1.68)	0.731	0.126	0.021
PEU5 = It would be easy for me to become skillful at using this tool., mean (std)	2.26 (1.58)	2.48 (1.69)	1.56 (0.79)	2.52 (1.81)	0.037	0.002	0.402
PEU6 = I would find this tool easy to use., mean (std)	2.22 (1.52)	2.46 (1.63)	1.56 (0.99)	2.33 (1.49)	0.011	<0.001	0.472
PSSUQ1 = Overall, I am satisfied with how easy it is to use this tool., mean (std)	2.47 (1.53)	2.58 (1.6)	2.09 (1.19)	2.67 (1.68)	0.415	0.125	0.469
PSSUQ2 = It was simple to use this tool., mean (std)	2.25 (1.48)	2.45 (1.62)	1.85 (1.28)	2.1 (1.0)	0.084	0.031	0.818
PSSUQ3 = I was able to complete the tasks and scenarios quickly using this tool., mean (std)	2.58 (1.3)	2.57 (1.36)	2.76 (1.37)	2.29 (0.85)	0.668	0.325	0.552
PSSUQ4 = I felt comfortable using this tool., mean (std)	2.79 (1.6)	2.75 (1.64)	2.56 (1.37)	3.33 (1.74)	0.534	0.482	0.090
PSSUQ5 = It was easy to learn to use this tool., mean (std)	2.25 (1.46)	2.48 (1.62)	1.94 (1.25)	1.86 (0.79)	0.07	0.159	0.432
PSSUQ6 = I believe I could become productive quickly using this tool., mean (std)	3.0 (1.57)	2.82 (1.43)	3.24 (1.44)	3.33 (2.15)	0.139	0.137	0.817
PSSUQ7 = The system gave error messages that clearly told me how to fix problems., mean (std)	4.1 (1.77)	3.44 (1.56)	5.35 (1.43)	4.71 (1.79)	<0.001	<0.001	0.135
PSSUQ8 = Whenever I made a mistake using the tool, I could recover easily and quickly., mean (std)	3.82 (1.73)	3.07 (1.32)	5.41 (1.58)	4.24 (1.61)	<0.001	<0.001	0.216
PSSUQ9 = The information (such as online help, on-screen messages, and other documentation provided with this tool was clear., mean (std)	3.42 (1.69)	2.9 (1.35)	4.91 (1.58)	3.1 (1.73)	<0.001	<0.001	0.353
PSSUQ10 = It was easy to find the information I needed., mean (std)	3.3 (1.7)	2.76 (1.35)	4.82 (1.83)	3.0 (1.26)	<0.001	<0.001	0.583
PSSUQ11 = The information was effective in helping me complete the tasks & scenarios., mean (std)	3.17 (1.64)	2.61 (1.27)	4.68 (1.55)	3.0 (1.61)	<0.001	<0.001	0.582
PSSUQ12 = The organization of information on the tool screens was clear., mean (std)	3.2 (1.76)	2.77 (1.56)	4.41 (1.94)	2.95 (1.32)	0.001	<0.001	0.742
PSSUQ13 = The interface of this tool was pleasant., mean (std)	2.53 (1.43)	2.54 (1.42)	2.53 (1.62)	2.52 (1.21)	0.859	0.639	0.752
PSSUQ14 = I liked using the interface of this tool., mean (std)	2.55 (1.46)	2.57 (1.41)	2.5 (1.66)	2.52 (1.36)	0.594	0.502	0.940
PSSUQ15 = This tool has all the functions and capabilities I expect it to have., mean (std)	4.12 (1.59)	3.6 (1.43)	4.74 (1.5)	5.24 (1.41)	<0.001	0.010	0.001
PSSUQ16 = Overall, I am satisfied with this tool., mean (std)	3.28 (1.48)	2.88 (1.37)	3.5 (1.42)	4.52 (1.29)	<0.001	0.282	<0.001
Could the tool give you any valuable information for the care path?, n (%)	108 (77.7%)	72 (85.71%)	24 (70.59%)	12 (57.14%)	0.007	0.343	0.022
Could the tool support and guide your work?, n (%)	120 (86.33%)	77 (91.67%)	29 (85.29%)	14 (66.67%)	0.041	0.782	0.010
Would the tool raise any legal or ethical considerations if it would be in clinical use?, n (%)	72 (51.8%)	52 (61.9%)	9 (26.47%)	11 (52.38%)	0.005	0.001	1.000
I would recommend this tool to colleagues?, n (%)	95 (68.35%)	70 (83.33%)	17 (50.0%)	8 (38.1%)	<0.001	0.011	0.002

Table S10. Post-hoc analysis of interaction effect between "open to adopting new technology"** and "have used prediction tools earlier"*** on the outcomes of perceived ease of use (PEU) and information quality (PSSUQ 7-12).

Outcome	Variable	Parameter	95% CI	P-value
PEU	Age	0.009	-0.024, 0.042	0.594
	Gender	-0.006	-0.041, 0.028	0.712
	Degree high	-0.023	-0.057, 0.012	0.199
	Open adopting new technologies	-0.103	-0.142, -0.065	<0.001*
	Have used prediction tools	-0.105	-0.247, 0.037	0.147
	(Open adopting new technologies) x (Have used prediction tools)	0.087	-0.056, 0.23	0.229
Information quality	Age	0.006	-0.029, 0.042	0.731
	Gender	-0.044	-0.082, -0.007	0.021*
	Degree high	0.004	-0.033, 0.041	0.833
	Open adopting new technologies	-0.071	-0.112, -0.03	0.001*
	Have used prediction tools	-0.121	-0.275, 0.033	0.121
	(Open adopting new technologies) x (Have used prediction tools)	0.171	0.017, 0.325	0.030*

Notes: *Significance level of Holm-Bonferroni-adjusted p-value <0.05

** How open are you to adopting new technologies in general in your healthcare practice? 1 (Not open at all), 2, 3, 4, 5 (Very open)

*** Have you used any decision support systems with predictions for patient trajectories? (no/yes)

Table S11. Post-hoc analysis of the effect of clinical experience on the additional questions measuring the clinical value of the tool. Model H1: Logistic regression models of years in clinical practice. Model H2: Logistic regression models of years of experience with older adults. The models were adjusted by position – physician, comfortable with using technology** and open adopting new technologies***.

Question	Model H1 Parameter	95% CI	p-values	Model H2 Parameter	95% CI	p-values
Q1: Could the tool give you any valuable information for the care path?	0.963	[0.929, 0.998]	0.038*	0.984	[0.946, 1.023]	0.418
Q2: Could the tool support and guide your work?	1.004	[0.963, 1.047]	0.846	1.040	[0.986, 1.096]	0.151
Q3: Would the tool raise any legal or ethical considerations if it would be in clinical use?	0.975	[0.946, 1.006]	0.108	1.001	[0.968, 1.034]	0.960
Q4: I would recommend this tool to colleagues?	0.987	[0.956, 1.02]	0.446	0.999	[0.963, 1.036]	0.956

Notes: *Significance level of Holm-Bonferroni-adjusted p-value <0.05

** How comfortable are you with using technology in general in your daily professional activities? 1 (Not comfortable at all), 2, 3, 4, 5 (Very comfortable)

*** How open are you to adopting new technologies in general in your healthcare practice? 1 (Not open at all), 2, 3, 4, 5 (Very open)
